# Hibernoma – two patients with a rare lipoid soft-tissue tumour

**DOI:** 10.1186/s12880-015-0046-3

**Published:** 2015-02-14

**Authors:** Dirk Daubner, Stephanie Spieth, Jessica Pablik, Klaus Zöphel, Tobias Paulus, Michael Laniado

**Affiliations:** Department of Neuroradiology, Carl Gustav Carus Medical School, University of Dresden, Fetscherstraße 74, 01307 Dresden, Germany; Department of Radiology, Carl Gustav Carus Medical School, University of Dresden, Fetscherstraße 74, 01307 Dresden, Germany; Department of Pathology, Carl Gustav Carus Medical School, University of Dresden, Fetscherstraße 74, 01307 Dresden, Germany; Department of Nuclear Medicine, Carl Gustav Carus Medical School, University of Dresden, Fetscherstraße 74, 01307 Dresden, Germany

**Keywords:** Hibernoma, Brown fat tissue, Soft-tissue tumour, Liposarcoma, MRI, ^18^ F-FDG-PET/CT

## Abstract

**Background:**

Hibernomas are rare benign soft-tissue tumours arising from brown fat tissue. Although imaging characteristics are not specific certain imaging features, common locations and patient demographics may suggest hibernoma as a differential diagnosis.

**Case presentation:**

We report on two 48-year-old male patients with hibernoma. The tumour presented with local swelling of the inguinal region in the first patient and was an incidental imaging finding in the second patient. Imaging included magnetic resonance imaging in both patients and computed tomography as well as ^18^ F-fluorodeoxyglucose positron emission tomography-computed tomography in the second patient. In both cases histological diagnosis was initially based on excisional and needle core biopsy, respectively. Complete surgical resection confirmed the diagnosis of hibernoma thereafter.

**Conclusion:**

In soft tissue tumours with fatty components hibernoma may be included into the differential diagnosis. Because of the risk of sampling errors in hibernoma-like tissue components of myxoid and well-differentiated liposarcoma, complete resection is mandatory. This article also reviews the current imaging literature of hibernomas.

## Background

Hibernomas are rare benign soft-tissue tumours arising from brown fat tissue [1-3]. The tumour was first described in 1906 by Merkel who named them “pseudolipoma” [4]. The name hibernoma was given by Gery in 1914 because of its similarity to brown fat in hibernating animals [5]. Hibernomas count for approximately 1.6% of all benign lipomatous tumours [6]. Until now, case reports and series with up to 170 patients were published [7]. Males are more often involved than females [7]. The typical age at presentation is between 30 and 50 years. Hibernomas are slowly growing, painless tumours, either presenting as palpable soft and mobile mass or as an incidental finding at imaging [3]. Due to the hypervascularity, hibernomas may sometimes appear with locally increased skin temperature. They can reach a size of up to 20 cm so that symptoms secondary to compression of adjacent structures may developed [7].

The aetiology of hibernomas is unknown. Molecular genetics show deletions respectively reciprocal translocation of chromosome 11 which encodes the tumour suppressor gene MEN 1 [8,9]. The development of hibernomas was initially thought to be related to areas of residual brown fat such as the interscapular area, axilla, chest wall, mediastinum or retroperitoneum. However, single case reports and a clinical-pathological study with 170 cases showed the thigh to be a preferential location with about 30% of the cases [7].

At histopathology, most hibernomas present as typical variant (82%) of which three cellular subtypes are differentiated (pale staining, mixed and eosinophilic cells). Myxoid variants (9%), lipoma-like variants (7%), and spindle cell variants (2%) of hibernoma are less common [3,7].

Except intrathoracic and retroperitoneal hibernomas, the tumours are usually well diagnosed with ultrasound showing smooth borders and hyperechoic signal. Colour duplex sonography and angiography often present a hypervascular tumour, with arteriovenous shunts in some cases. Due to the increased risk of bleeding, biopsy is not recommended in these cases. But often the diagnosis is not considered prior to biopsy in different imaging modalities, as presented in the two following cases [3,10,11].

In computed tomography (CT) and magnetic resonance imaging (MRI), hibernomas are well-circumscribed tumours, commonly located in subcutaneous tissue, skeletal muscle or intermuscular fascial planes. Common sites include thigh, peri- and interscapular region, axilla, neck, chest, abdominal cavity and retroperitoneum. The scalp, breast, scrotum and perirectal region, spine and bones are less frequently affected [3,7,12]. Hibernomas show relatively low attenuation in CT between the attenuation of subcutaneous fat and skeletal muscle. Intratumoural septa and a variable degree of enhancement may be seen [1,13,14]. In MRI, hibernomas may be slightly hypointense to fatty tissue on T1- and T2-weighted images and markedly hyperintense when fat suppressed T2-weighted inversion recovery sequences are applied. Elongated and branching vessels within the tumour are typically seen as flow void phenomena in MRI. Nevertheless, lack of intratumoural vessels does not exclude hibernoma [2,3,10]. After intravenous contrast agent administration strong enhancement of intratumoural blood vessels and homogeneous or heterogeneous tumour enhancement may be observed [10]. In ^18^ F-fluorodeoxyglucose (FDG) positron emission tomography-computed tomography (PET/CT) high uptake is measured in hibernomas due to the metabolic activity of brown fat. Therefore, differentiation from malignant tumours is difficult [3,10,15,16]. Compared to liposarcomas, hibernomas often show higher standardized uptake values (SUVs) and change of FDG avidity in follow-up. However, these criteria are not reliable enough to make the diagnosis based on imaging alone [16,17].

Hibernomas are benign tumours without a risk of malignant transformation or metastases [7,18]. As soon as the diagnosis is confirmed, no surgical removal or other treatment is required in asymptomatic hibernoma [16]. However, histological features similar to hibernomas are described in atypical fatty tumours as well as in myxoid and well-differentiated liposarcomas. Consequently, biopsy proven diagnosis of hibernoma does not exclude hibernoma-like differentiation in myxoid or well-differentiated liposarcomas and complete excision is indicated. Incomplete resection of hibernoma carries the risk of recurrence, whereas complete resection cures the disease [7,19].

## Case presentation

### Case 1

**Figure 1:**
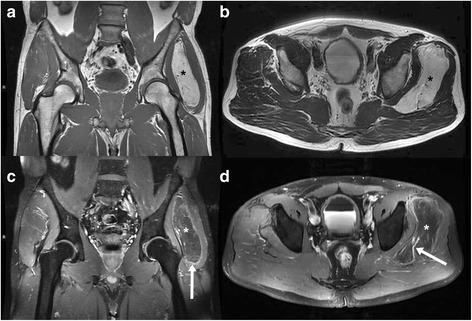
**48-year-old man with hibernoma of the left upper leg.** Tumour between the gluteus medius and minimus muscles (star in image 1 **a-d**). MRI shows an isointense tumour compared to fat with slight rim enhancement (arrow in **c**) and a prominent vessel within the mass (arrow in **d**). **a** plain T1-weighted-TSE (TR 819 ms/ TE 11 ms), **b** plain T2-weighted-TSE (TR 5050 ms/ TE 96 ms), **c** contrast enhanced fat saturated T1-weighted-TSE (TR 895 ms/ TE 11 ms), **d** contrast enhanced fat saturated T1-weighted-TSE (TR 680 ms/ TE 11 ms).

A 48-year-old male patient was admitted to our hospital with a palpable soft-tissue swelling at the left upper leg extending to the groin. The tumour was slowly growing since approximately 6 months. MRI was performed (Siemens Magnetom Verio®, 3.0 Tesla, body-array-coil, Erlangen, Germany) showing a 4 × 10 × 12 cm measuring mass between the gluteus medius and minimus muscles which was isointense to fat in all sequences. The tumour extended from the greater trochanter of the femur to the anterior superior iliac spine (Figure 1). After intravenous administration of contrast medium (15 ml Magnevist®, 0.5 mmol/ml, Bayer Vital GmbH, Leverkusen, Germany) slight rim enhancement of the tumour (arrow in Figure 1c) and a prominent vessel within the tumour (arrow in Figure 1d) were seen. Based on imaging findings, the working diagnosis was liposarcoma. The case was discussed in the multidisciplinary sarcoma board of our comprehensive cancer center. As a result, diagnostic excision was suggested which was performed without complications.

**Figure 2:**
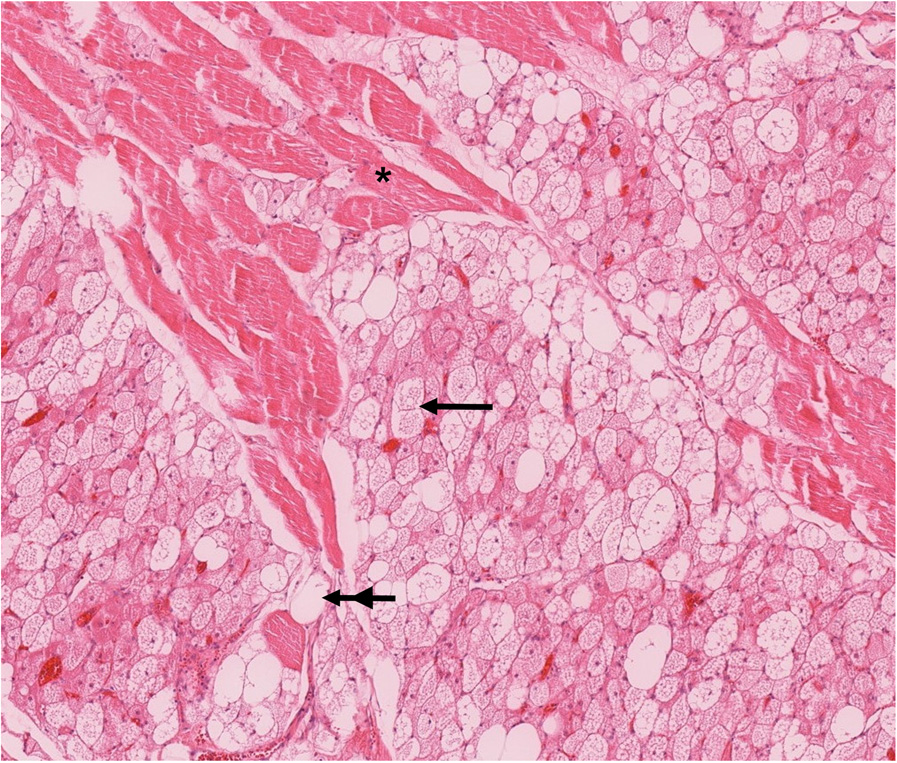
**Tumour, composed of multivacuolated eosinophilic (arrow) and pale cells (double arrow), dissecting between skeletal muscle fibers (asterisk).** H & E stain, original magnification 10x.

Histopathology of the specimen (2 × 1.2 × 1 cm) revealed an intramuscular lipomatous neoplasia, complying with the diagnosis of the pale cell subtype of typical hibernoma (Figure 2). In-toto excision of the encapsulated hibernoma followed two weeks later. Histopathology confirmed the diagnosis of hibernoma and liposarcoma could be excluded. Postsurgery MRI follow-up after one year proved that no recurrence has occurred.

### Case 2

A 48-year-old male patient presented in our emergency department with upper abdominal pain, elevated liver enzymes and laboratory parameters of cholestasis. Acute cholecystitis was suspected and abdominal ultrasound was performed. Due to overlying bowel gas the gallbladder could not be examined completely. Consequently, abdominal CT (Siemens Somatom Definition AS + ®, Erlangen, Germany, collimation 2 × 64 × 0.6 mm, current 129 mA, tube voltage 120 kV) with both intravenous (90 ml Solutrast® 300 mg I/ml, Bracco Imaging Deutschland GmbH, Konstanz, Germany; imaging delay 65 sec.) and oral contrast administration (1000 ml H_2_0 + 30 ml Gastrolux® 370 mg I/ml, Sanochemia Diagnostics Deutschland GmbH, Neuss, Germany) was performed to exclude gallbladder perforation. CT showed increased enhancement of the gallbladder wall and pericholecystitic infiltration of fatty tissue compatible with acalculous cholecystitis. There was no dilatation or wall thickening of the intra- or extrahepatic bile ducts. Endosonography was performed thereafter, showing a single stone in the middle part of the common bile duct, which was treated with endoscopic retrograde cholangiopancreatography.

**Figure 3:**
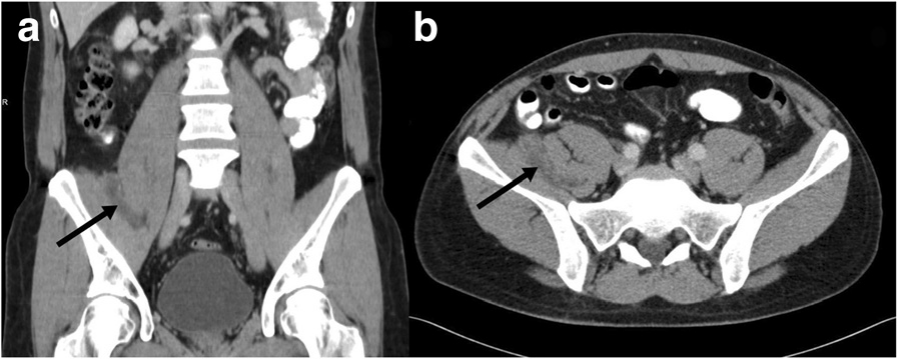
**48-year-old man with right-sided retroperitoneal hibernoma.** Tumour between the psoas and iliac muscles (arrows). Contrast enhanced CT shows minimal heterogeneous enhancement of the tumour. **a** coronal reconstruction, **b** axial slice.

**Figure 4:**
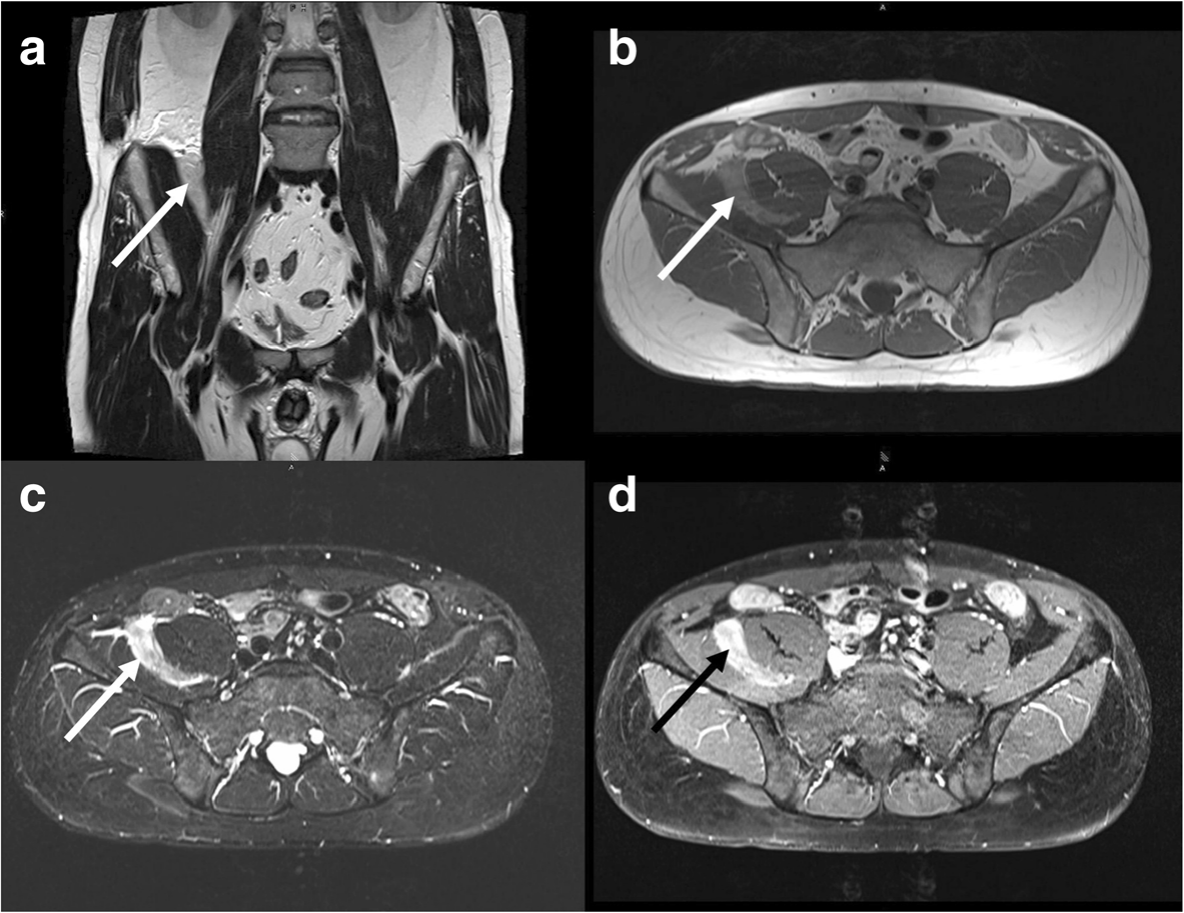
**MRI of the same patient as shown in Figure** **3** **.** The tumour (arrows) is slightly hypointense compared to mesenteric fat in T2- (**a**, TSE, TR 5040 ms/ TE 137 ms) and T1-weighted images (**b**, TSE, TR 651 ms/ TE 11 ms). The T2-weighted TIRM sequence **(c)** shows marked hyperintensity of the mass (**c**, TR 5000 ms/ TE 74 ms/ TI 170 ms). After administration of contrast medium heterogeneous enhancement occurs (**d**, fat saturated TSE, TR 600 ms/ TE 11 ms).

**Figure 5:**
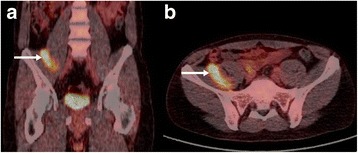
^**18**^ **F-FDG-PET/CT of the same patient as shown in Figure** **3** **and** **4** **demonstrates increased FDG-activity of the mass (arrows).** CT was performed in low-dose technique (9/97 mAs, 100/120 kV). **a** coronal reconstruction, **b** axial slice.

CT incidentally revealed a 5.5 × 1.5 × 5.5 cm measuring retroperitoneal tumour located between the right psoas and iliac muscles (Figure 3). The tumour showed mean density values between 10–15 Hounsfield Units (HU). For further diagnostic work-up MRI of the abdomen was performed (Siemens Magnetom Verio®, 3.0 Tesla, body-array-coil, Erlangen, Germany). Turbo inversion recovery magnitude (TIRM) sequences revealed a hyperintense tumour that was slightly hypointense in T1- and T2-weighted sequences compared to mesenteric fat. After administration of intravenous contrast agent (15 ml Magnevist®, 0,5 mmol/ml, Bayer Vital GmbH, Leverkusen, Germany) there was a strong heterogeneous enhancement. Based on CT and MRI the working diagnosis was a potentially malignant mesenchymal tumour (Figure 4). Therefore, PET/CT (Siemens Biograph 16®, Knoxville, USA, collimation 16 × 1.5 mm, tube currency 9/97 mAs, tube voltage 100/120 kV) with ^18^ F-FDG (5 MBq/kg body weight) was performed which showed an increased accumulation of ^18^ F-FDG (SUV_max_ 12.3) within the tumour 60 minutes after injection. ^18^ F-FDG-PET/CT further supported the working diagnosis of a malignant tumour (Figure 5).

**Figure 6:**
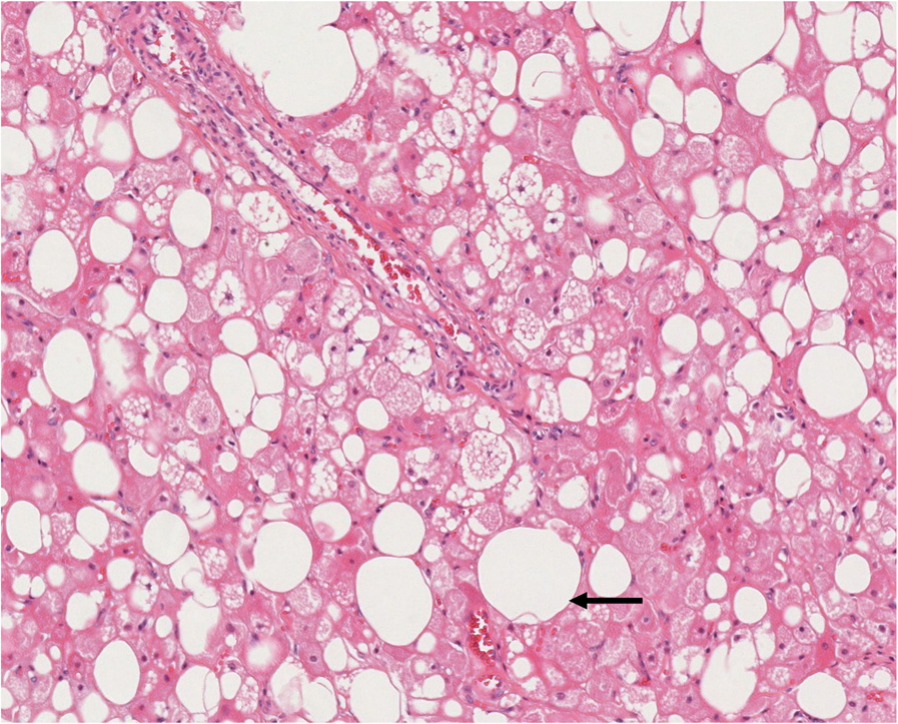
**Surgical specimen of hibernoma with univacuolar white fat cells (arrow).** H & E stain, original magnification 15× .

Histological examination was performed after CT-guided biopsy of the soft-tissue lesion (Quick Core Biopsy Needle Sets QCS-18-15-20 T, Cook Medical Inc., Bloomington, USA). Histological analysis yielded the pale cell subtype of a typical hibernoma (Figure 6). After discussion in the multidisciplinary sarcoma board of the comprehensive cancer center at our hospital elective tumour excision was performed 2 months after biopsy. The final histological diagnosis of the surgical specimen confirmed the presumptive diagnosis of the biopsy.

## Conclusion

Hibernomas are benign, well-circumscribed, slowly growing and well vascularised soft-tissue tumours containing brown fat [1-3,7]. MRI and ^18^ F-FDG-PET may provide imaging findings suggestive of hibernoma. These findings include hypointensity to fatty tissue on T2-weighted images and hyperintense signal intensity on TIRM images. Large intratumoural blood vessels, heterogeneous contrast enhancement and strong FDG uptake are further imaging signs [2,3,10,15,16].

The differential diagnosis of lipomatous soft-tissue tumours is broad and comprises benign (e. g. lipoma, hemangioma, angiolipoma) and malignant (e. g. liposarcoma) lesions [1,14,20]. Lipomas are characterized by homogeneous adipose tissue which can show thin septa. There is no contrast enhancement within lipomas, although infections, infarctions and necrosis as well as muscle fibres, intratumoural vessels und fibrotic septa may hamper the diagnosis [21]. Dependent on fat content, haemangiomas and angiolipomas show hypo- to hyperintense signal on T1-weighted images and hyperintense signal on T2-weighted images with intrinsic vessels. Haemangiomas can also contain calcifications similar to phlebolits [10]. Imaging characteristics of liposarcomas depends on the histological differentiation. Well-differentiated liposarcomas have thickened septa (>2 mm) as well as areas of T2-hyperintensity. Enhancement can be absent or circumscribed [10,20,21]. The signal intensity on T1-weighted images is often similar to subcutaneous fat. Moreover, large vessels are lacking which helps to distinguish liposarcomas from hibernomas. Poorly differentiated liposarcomas are rarely confounded with hibernomas due to the low fat content [20,21].

However, despite the use of multimodal imaging, the radiologic diagnosis of hibernomas is difficult and differentiation from well-differentiated liposarcomas remains a challenge even after biopsy. Therefore, complete surgical resection is highly recommended. A summary table of hibernomas is shown in Table 1.

**Table 1:** **Summary table of hibernomas**

Aetiology	Unclear, deletion respectively reciprocal translocation of chromosome 11
Incidence	Approximately 1.6% of all benign lipomatous tumours
Gender distribution	Slight male predominance
Average age	3rd to 4th decade of life
Risk factors	Not known
Therapy	Complete excision of symptomatic hibernoma or unclear diagnosis
Prognosis	Benign tumour without malignant transformation or metastatic spread
	No recurrence after complete excision, possible recurrence in cases of incomplete excision
Imaging characteristics	Well-defined, richly vascularized soft tissue tumour with a density between subcutaneous fat and skeletal muscle in CT, respectively slightly hypointense to subcutaneous fat in T1w and T2w MR images, hyperintense in TIRM, heterogenous to homogenous enhancement, high ^18^ F-FDG uptake and change of FDG avidity in follow-up

### Consent

Written informed consent was obtained from both patients for publication of this Case report and any accompanying images. A copy of the written consents is available for review by the Editor of this journal.

## Abbreviations

CT: Computed tomography
MRI: Magnetic resonance imaging
FDG: Fluorodeoxyglucose
PET: Positron emission tomography
TIRM: Turbo inversion recovery magnitude
HU: Hounsfield units
SUV: Standardized uptake value
